# Analysis of the Effect of Phacoemulsification and Intraocular Lens Implantation Combined With Trabeculectomy on Cataract and Its Influence on Corneal Endothelium

**DOI:** 10.3389/fsurg.2022.841296

**Published:** 2022-02-18

**Authors:** Bin Wang, Liqin Tang

**Affiliations:** Department of Ophthalmology, The First People's Hospital of Fuyang District, Hangzhou, China

**Keywords:** cataract, phacoemulsification, intraocular lens implantation, trabeculectomy, corneal endothelium

## Abstract

**Objective:**

This study aimed to discuss the effect of phacoemulsification and intraocular lens implantation (PHACO + IOL) combined with trabeculectomy (TRAB) on cataracts and its influence on the corneal endothelium.

**Methods:**

We selected 120 cataract patients admitted to our hospital from January 2018 to January 2021. According to different surgical methods, they were divided into the control group and the observation group. The observation group was treated with PHACO + IOL combined with TRAB, the control group only received PHACO. The clinical effect, ophthalmic-related parameters, corneal endothelium, complications, the satisfaction of the two groups were observed.

**Results:**

The total effective rate and total satisfaction rate of the observation group were significantly higher than the control group (*P* < 0.05). One month after the operation, the vision and central anterior chamber depth of the observation group were higher than those of the control group, and intraocular pressure (IOP) was lower than that of the control group (*P* < 0.05). One month after the operation, the corneal endothelial cell area and cell density in the observation group were not significantly different from those before operation (*P* > 0.05). There was no significant difference in the total incidence of complications between the two groups (*P* > 0.05).

**Conclusion:**

This study concluded that PHACO + IOL combined with TRAB has a good curative effect in the treatment of cataracts, which can improve the patients' vision and IOP, keep the functional integrity of corneal endothelial cells, and does not increase the occurrence of complications, the patients' satisfaction is high.

## Introduction

A cataract is the first kind of blinding eye disease in China and even in the whole world. Usually, the metabolic disorder of the patient's eye lens leads to degeneration of lens protein, which leads to intraocular lens opacity (1). Cataracts mostly occur in middle-aged and elderly people over 50 years old, which can be caused by age, decreased immunity, radiation, drugs, poisoning, and other factors. However, there are cases of cataracts at birth, and congenital cataracts are mostly influenced by genetic factors, which may be closely related to maternal nutrition deficiency, metabolic disorder, bacterial virus infection during pregnancy, a large amount of harmful radiation, and other factors (2, 3). The main symptoms of cataracts are decreased vision, a spot-shaped fixed shadow in the visual field, one eye diplopia or polyopia, refractive changes, visual distortion, and finally color vision changes, resulting in blindness, these symptoms seriously affect the daily life of patients (4). According to reports, the incidence of cataracts is 60–70% during the period of 50–60 years old, and the incidence of cataracts over 70 years old can reach 80%. At the same time, it is estimated that cataract is the main cause of vision decline, accounting for about 54.20%, and blindness accounts for 17.8%. This disease has become the leading cause of blindness and visual disability in the world (5). With the development of cataract, the patient's lens enters the expansion period, its volume increases, and its anterior-posterior diameter becomes thicker, which increases the contact area between lens and iris, leading to the increase of the resistance of the posterior chamber aqueous drainage to the anterior chamber, resulting in the pupillary block. When the pressure of the posterior chamber cannot overcome the pupillary block, the peripheral iris swells obviously, leading to the stenosis of the anterior chamber angle and even the occlusion of the anterior chamber angle, which leads to glaucoma (6, 7). Therefore, effective treatment of cataract patients is needed in time.

At present, for cataract patients, the therapeutic effect of drugs is not obvious, so the operation has become the main treatment of cataracts. Phacoemulsification intraocular lens implantation (PHACO + IOL) and trabeculectomy (TRAB) are effective methods to treat cataracts, but the single treatment method is difficult to achieve the desired goal (8). In recent years, the choice of surgical scheme for cataract patients is still controversial. With the continuous improvement of medical equipment and medical technology, one-time combined operation is more and more accepted by ophthalmologists. In this study, 120 patients with cataracts were selected as the observation object, O + IOL + TRAB treatment was performed, and the clinical efficacy of this operation for cataracts was discussed. The specific report was as follows.

## Materials and Methods

### Research Object

We selected 120 cataract patients admitted to our hospital from January 2018 to January 2021. According to different surgical methods, they were divided into the control group (58 cases, 81 eyes) and the observation group (62 cases, 85 eyes). There were 31 men and 27 women in the control group, with an average age of (59.34 ± 4.88) years. The average disease duration was (5.2 ± 0.6) months. Emery nuclear hardness classification: 26 eyes were grade I, 29 eyes were grade II, and 26 eyes were grade III. There were 32 men and 30 women in the observation group, with an average age of (59.6 ± 4.71) years. The average disease duration was (5.1 ± 0.7) months. Emery nuclear hardness classification: 27 eyes were grade I, 30 eyes were grade II, and 28 eyes were grade III. General data of the two groups were balanced and comparable (*P* > 0.05). Inclusion criteria include consistent with the diagnosis of cataract, lens opacity can be seen in pupil area, including vacuoles, water cracks, lamellar separation, wheel-width opacity, wedge-shaped opacity, nuclear opacity, and posterior capsular opacity, etc. Exclusion criteria include combined with other eye diseases, high myopia, having a history of an eye operation, complicated with serious organic diseases, and there was eye trauma recently.

### Research Methods

#### Preoperative Treatment

Different intraocular pressure-lowering measures were taken according to the intraocular pressure of the patients when they were admitted to the hospital. The routine treatment was as follows: The two groups were given pilocarpine eye drops, a mydriatic agent, 10 min/time, 3–5 times in a row. Timolol maleate eye drops and tobramycin dexamethasone eye drops were used to control intraocular pressure (IOP) in affected eyes once.

#### Operation Technique

The observation group was treated with PHACO + IOL combined with TRAB. First of all, Eye surface anesthesia was carried out. PHACO + IOL: A conjunctival flap based on the dome was made above the limbal of the cornea, and a scleral flap with a thickness of 4 × 3 mm and a thickness of 1/2 of the sclera was made after the sclera was burned to stop bleeding. The flap was divided into transparent corneas of 1 mm, and a continuous annular capsulorhexis with a diameter of 5–6 mm was cut in the middle of the anterior capsule of the lens with a capsulorhexis tool. Water was separated and stratified, then, the hard lens nucleus was crushed into a chylous shape by Universal II phacoemulsification machine (Alcon, USA), and then sucked out. The free cortex was sucked out, then the cortex adhered to the capsule was removed. Afterward, the capsule was polished and a viscoelastic agent was inserted. The annular capsulorhexis was enlarged and the IOL was inserted into the capsule and adjusted to the appropriate position. Then TRAB was performed: 1 mm × 3 mm trabecula and the surrounding 1/3 iris were removed, the scleral flap was sutured intermittently with 10-0 nylon thread, viscoelastic agent in the anterior chamber was sucked out, the anterior chamber was formed, and filtration was checked at the same time; the conjunctiva and fascia were reduced and sutured continuously with 10-0 nylon thread. The control group only received PHACO operation, and the operation method was the same as the observation group.

#### Postoperative Treatment

Indomethacin was given orally, 25 mg/time, 3 times/day; acetazolamide was given orally, 250 mg/time, 2 times/day; tobramycin dexamethasone eye drops and pilocarpine eye drops were given, 1 time/day, and the medication lasted for 5–7 days.

### Observation Index

(1) The curative effect was determined 1 month after the operation. The criterion of curative effect: Markedly effective: the lesion disappeared, without discomfort in the eyes; Effective: the symptoms improved, and the patient felt that the symptoms improved; Invalid: the disease has not improved, and even worsened. Total effective rate = markedly effective rate + effective rate.

(2) Before and 1 month after the operation, the vision was measured by comprehensive refractometer, IOP was measured by non-contact tonometer, and ophthalmic related parameters such as central anterior chamber depth (cACD), lens thickness (LT), and axial length (AL) were measured by A-mode ultrasound, each eye was tested 10 times and the average value was taken.

(3) Before and 1 month after the operation, at the workstation equipped with image processing, the images of the central living area of corneal endothelial cells of patients were obtained by using a non-contact corneal endothelial cell microscope, and the corneal endothelial cell area (ECA) and corneal endothelial cell density (CD) were measured.

(4) The complications such as the shallow anterior chamber, anterior chamber hemorrhage, cellulose exudation of iris, corneal edema, corneal endothelial fold, and anterior chamber inflammation were recorded after the operation.

(5) The satisfaction questionnaire prepared by the undergraduate department was used to evaluate patients' satisfaction from the aspects of visual condition, clarity, comfort, stability, and other indicators. The Likert 5 method was used to score, and the score of very satisfaction was 90–100 points, satisfaction was 70–89 points, and dissatisfaction was < 70 points. Total satisfaction = very satisfaction rate + satisfaction rate. The self-made questionnaire in our hospital had good reliability, with a reliability of .81.

### Statistical Methods

For data processing, SPSS 22 software, Armonk, NY; IBM Corporation was used, and the enumeration data were expressed as rate (%), χ^2^ test was used for comparison. Measurement data were expressed as ($\bar{x}$ ± s), *t*-test was used for comparison. *P* < 0.05 indicated that the difference was statistically significant.

## Results

### Clinical Effect of Two Groups

The total effective rate of the observation group was 98.82%, which was significantly higher than that of the control group (87.65%) (*P* < 0.05) (Table 1).

**Table 1:** Clinical effect of two groups (*n*, %).

**Group**	**Eye number**	**Markedly effective rate**	**Effective rate**	**Invalid rate**	**Total effective rate**
Control group (*n* = 58)	81	51 (62.96%)	20 (24.69%)	10 (12.35%)	71 (87.65%)
Observation group (*n* = 62)	85	59 (69.41%)	25 (29.41%)	1 (1.18%)	84 (98.82%)
*χ^2^* value					8.362
*P* value					0.004

### Ophthalmic Related Parameters of Two Groups

**Figure 1:**
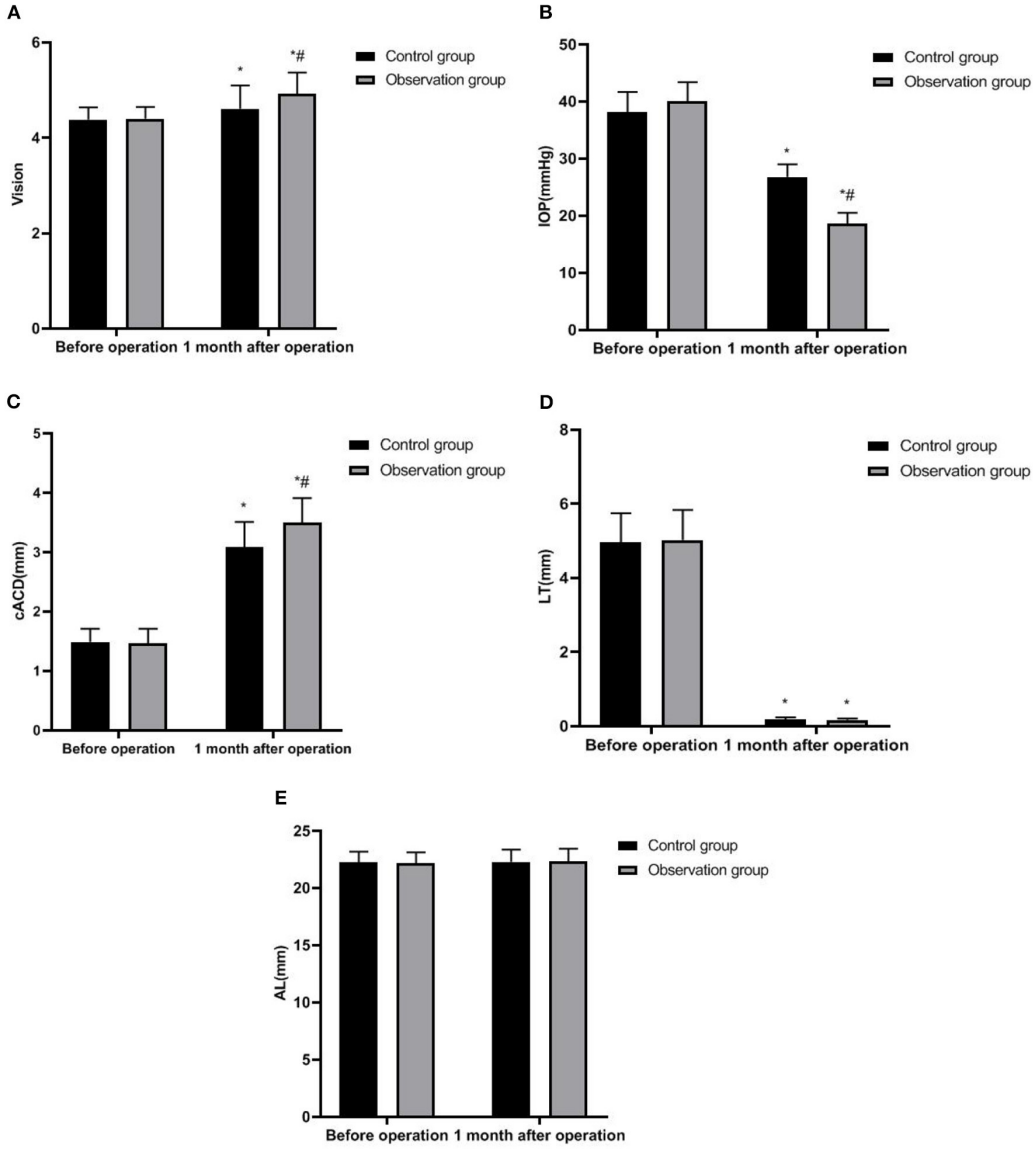
Ophthalmic-related parameters of two groups. **(A)** Vision; **(B)** IOP; **(C)** cACD; **(D)** LT; and **(E)** AL. Compared with before operation, *P < 0.05; Compared with the control group, ^#^*P* < 0.05.

One month after the operation, the vision, IOP, cACD, and LT of the two groups were significantly different from those before operation (*P* < 0.05). One month after the operation, the vision and cACD of the observation group were higher than those of the control group, and IOP was lower than that of the control group (*P* < 0.05) (Figure 1).

### Corneal Endothelium of Two Groups

**Figure 2:**
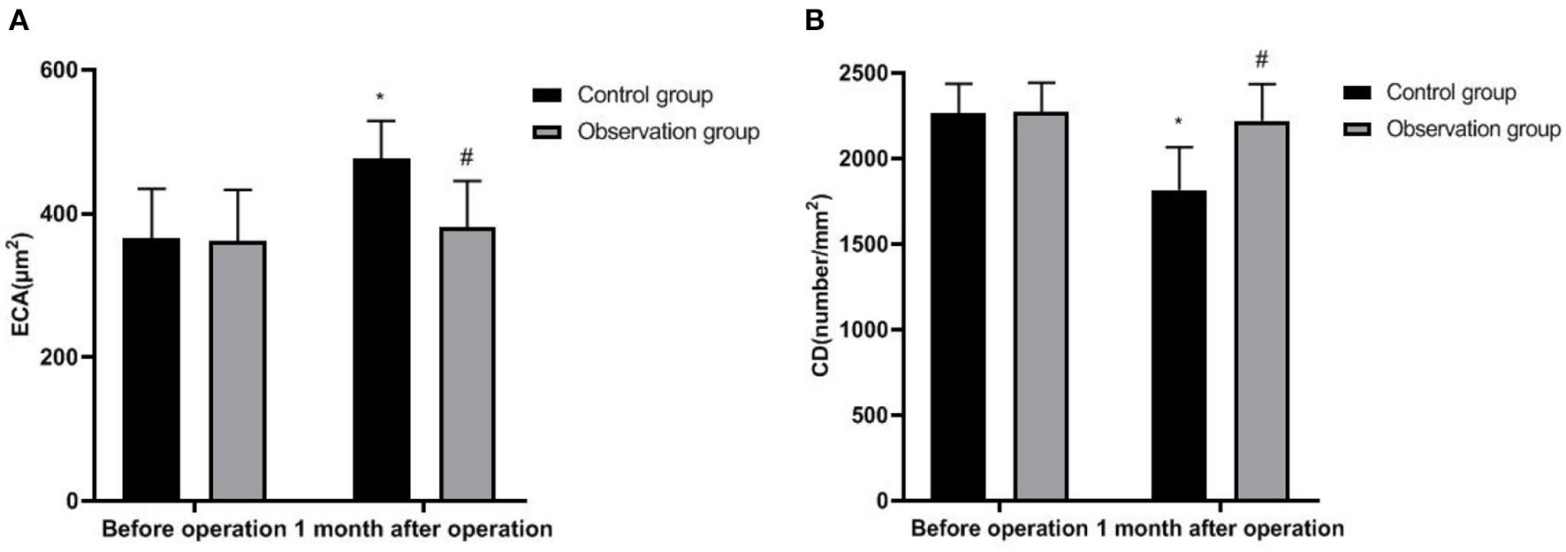
Corneal endothelium of two groups. **(A)** ECA and **(B)** CD. Compared with before operation, *P < 0.05; Compared with the control group, ^#^*P* < 0.05.

One month after the operation, the ECA and CD in the observation group were not significantly different from those before the operation (*P* > 0.05). ECA in the observation group was lower than the control group, the CD was higher than the control group (*P* < 0.05) (Figure 2).

### Complications of Two Groups

There was no significant difference in the total incidence of complications between the two groups (*P* > 0.05) (Table 2).

**Table 2:** Complications in two groups (*n*, %).

**Group**	**Eye number**	**Shallowanteriorchamber**	**Anteriorchamberhemorrhage**	**Celluloseexudation ofiris**	**Cornealedema**	**Other**	**Totalincidencerate**
Control group (*n* = 58)	81	2 (2.47%)	1 (1.23%)	2 (2.47%)	2 (2.47%)	2 (2.47%)	9 (11.11%)
Observation group (*n* = 62)	85	2 (2.35%)	0 (0.00%)	1 (1.18%)	1 (1.18%)	0 (0.00%)	4 (4.71%)
*χ^2^* value							2.357
*P* value							0.125

### The Satisfaction Rate of Two Groups

The total satisfaction rate of the observation group was 88.24%, which was significantly higher than that of the control group (75.31%) (*P* < 0.05) (Table 3).

**Table 3:** The satisfaction rate of two groups (*n*, %).

**Group**	**Eye number**	**Very satisfactory rate**	**Satisfaction rate**	**Dissatisfaction rate**	**Total satisfaction rate**
Control group (*n* = 58)	81	40 (49.38%)	21 (25.93%)	20 (24.69%)	61 (75.31%)
Observation group (*n* =62)	85	46 (54.12%)	29 (34.12%)	10 (11.76%)	75 (88.24%)
*χ^2^* value					4.681
*P* value					0.031

## Discussion

With the aging of the population, the number of cases of visual impairment caused by cataracts is increasing. Cataract leads to the closure of the angle of the chamber, the thickness of the lens increases in the elderly, and the relaxation of the suspensory ligament leads to the relative forward movement of the lens (9, 10). How to treat these patients effectively has become one of the key problems to be solved urgently in clinical ophthalmology. In recent years, cataract operation has made rapid development, and different surgical methods have different therapeutic effects, which significantly improves the recovery rate of patients.

Phacoemulsification (PHACO) is a surgical method for cataract treatment by using phacoemulsification instruments. The therapeutic principle of this technology is to establish surgical access in the incision of cornea or sclera through the phacoemulsification handle, then form a capsulorhexis, and use the ultrasonic needle to generate certain energy in the eyes, so as to crush the hard lens into chylous shape, and then suck out the crushed lens and free cortex with the help of perfusion suction system (11, 12). This operation can deepen the anterior chamber, enlarge the angle of the chamber, improve the pupillary block caused by the lens, and solve the anatomical factors such as lens thickening, relative forward movement of the lens position, closure of the remaining angle of the chamber in cataract patients, thus rapidly reducing the IOP of the patients (13). During the implementation of PHACO, ultrasound itself can cause the secretion function of the ciliary body to decline, and the placement of viscoelastic agent forms a passive separation effect on the corner adhesion. At the same time, it has the advantages of good sealing of surgical incision, short time, light postoperative reaction, quick recovery of vision, etc., which has received extensive attention from clinicians (14–16). However, giving cataract patients a single surgical procedure may not meet their ideal needs. The implementation of PHACO cannot change the structure of the highly pleated iris, cannot effectively solve the iris bulge, and the iris still adheres to the ciliary body. Moreover, the application of PHACO preoperative intraocular hypertension drugs will lead to the destruction of the ocular trabecular structure of patients, resulting in the continuous increase of IOP and the impaired function of corneal endothelial cells and other adverse changes. Finally, cataract patients may need a second operation, which not only increases the iatrogenic injury of patients but also increases the medical expenses (17).

In recent years, PHACO + IOL combined with TRAB has become the focus of clinical ophthalmology. PHACO + IOL is a surgical method of implanting an intraocular lens with thickness <1.0 mm in the capsular bag to replace the human lens with a thickness of about 5.0 mm. After the operation, the anterior chamber volume can be improved, the cACD can be significantly deepened, and the contact plane between pupil margin and lens can be moved backward, thus reducing pupil block, widening the angle of the chamber, and reducing the outflow resistance of aqueous humor, resulting in effective control of vision and IOP compared with before operation (18). TRAB was first established by Cairns's team. It is to re-establish an extra-ocular drainage channel of aqueous humor at the corneal limbus, so that part of aqueous humor in the trabecular meshwork can be drained out of the filtering bleb, which has a positive effect on lowering IOP (19). In the operation of PHACO + IOL combined with TRAB, the operator directly removes the sinus trabecular tissue and the surrounding iris tissue after implantation of intraocular lens, and in the process of cataract extraction, with the help of the operation of the ultrasonic emulsifying machine, the trabecular meshwork deposits can be removed, and the filtering effect of trabecular meshwork can be improved. The application of viscoelastic agent can compress iris blood vessels, reduce the pressure of the anterior chamber, improve the structure of the anterior chamber, adjust the lens position, further relieve pupillary block, maintain the stability of the anterior chamber, and make the IOP after operation return to normal and effectively improve (20, 21). PHACO+IOL combined with TRAB has many advantages when applied to cataract patients. The operation can be completed at one time, which avoids eye trauma caused by multiple operations and reduces the chance of tissue bleeding; It can shorten the treatment period, relieve the pain of patients and save the cost of operation; Compared with the single operation, the combined operation can improve the operation quality, and achieve the better long-term effect of intraocular pressure control. It can relieve many glaucoma factors at one time (22, 23). In this study, the vision, IOP, cACD, and LT of patients in PHACO + IOL combined with the TRAB group were significantly better than those before the operation, and the total effective rate, vision, IOP, and cACD were significantly better than those in a single operation, and the patients had higher satisfaction. This is roughly consistent with the research results of Wang's team (24).

The corneal transparency of patients has an important impact on the recovery of vision in cataract patients after operation, and the structural and functional integrity of corneal endothelial cells is the main condition to ensure corneal transparency. Choi's team reported that corneal endothelial cells can only be repaired by normal cell expansion after injury, but when CD <1,000 cells /mm^2^, the damage rate of corneal endothelial cells will exceed its function compensation rate, resulting in corneal endothelial cells being unable to maintain normal function (25). Our research results have shown that the ECA of the observation group was lower than that of the control group, and the CD was higher than that of the control group, which revealed that cataract patients who only implement PHACO have more serious damage to corneal endothelial cells, and PHACO itself can cause damage to corneal endothelial cells. The degree of damage is influenced by factors such as ultrasonic energy and time, lens nucleus hardness, operator's operation proficiency, and so on, and may be related to the duration of high intraocular pressure. At the same time, we observed that there was no significant difference in ECA and CD in the observation group after operation compared with that before operation. The scheme of the PHA + IOL + TRAB combined operation is quite perfect. By suctioning out the cloudy lens, implanting intraocular lens, cutting out the trabecular sinus tissue, and other operations, the vision and IOP of patients can be improved, and the corneal endothelial cells of patients are less damaged, and the functional integrity of corneal endothelial cells can be effectively maintained. In addition, in this study, the incidence of complications of the combined operation scheme is not high, only 4.71%, and there was no corneal endothelial fold and anterior chamber inflammation, which had good safety. TRAB drainage of aqueous humor from the anterior chamber to subconjunctival space and covering the drainage opening with scleral lamina can limit the excessive outflow of aqueous humor and reduce complications such as shallow anterior chamber and hyphema after the operation. One-time PHACO + IOL + TRAB operation can avoid transient ocular hypertension caused by the staged operation, prevent optic nerve damage caused by multiple intraocular operations and reduce complications.

## Conclusion

From the above results, it can be seen that PHACO + IOL combined with TRAB has a good curative effect in the treatment of cataracts, which can improve the patients' vision and IOP, keep the functional integrity of corneal endothelial cells, and does not increase the occurrence of complications, the patients' satisfaction is high. The success of the operation depends on many factors, such as the damage degree of the trabecular meshwork, the extent, and duration of angle-closure, etc. Generally speaking, the shorter the closure time of atrial Angle adhesion, the better the prognosis of operation. Therefore, the selection of surgical timing is also very important, and the appropriate surgical timing is conducive to improving the success rate of operation.

## Data Availability Statement

The original contributions presented in the study are included in the article/supplementary material, further inquiries can be directed to the corresponding author.

## Ethics Statement

The studies involving human participants were reviewed and approved by the Ethics Committee of The First People's Hospital of Fuyang District, Hangzhou. The patients/participants provided their written informed consent to participate in this study.

## Author Contributions

BW was mainly responsible for the design of the research and the writing of the thesis. LT was mainly responsible for the implementation of the research project. All authors contributed to the article and approved the submitted version.

## Conflict of Interest

The authors declare that the research was conducted in the absence of any commercial or financial relationships that could be construed as a potential conflict of interest.

## Publisher's Note

All claims expressed in this article are solely those of the authors and do not necessarily represent those of their affiliated organizations, or those of the publisher, the editors and the reviewers. Any product that may be evaluated in this article, or claim that may be made by its manufacturer, is not guaranteed or endorsed by the publisher.

## References

[1] Sangwan VS Gupta S Das S. Cataract surgery in ocular surface diseases: clinical challenges and outcomes. Curr Opin Ophthalmol. (2018) 29:81–7. 10.1097/ICU.0000000000000441 29210839

[2] Fukuoka H Afshari NA. The impact of age-related cataract on measures of frailty in an aging global population. Curr Opin Ophthalmol. (2017) 28:93–7. 10.1097/ICU.0000000000000338 27820747

[3] Self JE Taylor R Solebo AL Biswas S Parulekar M Dev Borman A. Cataract management in children: a review of the literature and current practice across five large UK centres. Eye (Lond). (2020) 34:2197–218. 10.1038/s41433-020-1115-6 32778738 PMC7784951

[4] Avetisov KS Ivanov MN Yusef YN Yusef SN Aslamazova AE Fokina ND. [Morphological and clinical aspects of anterior capsulotomy in femtosecond laser-assisted cataract surgery]. Vestn Oftalmol. (2017) 133:83–8. 10.17116/oftalma2017133483-88 28980571

[5] Evans J Mwangi N Burn H Ramke J. Equity was rarely considered in Cochrane Eyes and Vision systematic reviews and primary studies on cataract. J Clin Epidemiol. (2020) 125:57–63. 10.1016/j.jclinepi.2020.04.024 32389807

[6] Agrawal S Fledderjohann J Ghosh S. Risk factors for self-reported cataract symptoms, diagnosis, and surgery uptake among older adults in India: Findings from the WHO SAGE data. Glob Public Health. (2021) 16:1771–85. 10.1080/17441692.2020.1836246 33091324

[7] Sarkisian SR Jr Radcliffe N Harasymowycz P Vold S Patrianakos T Zhang A. Visual outcomes of combined cataract surgery and minimally invasive glaucoma surgery. J Cataract Refract Surg. (2020) 46:1422–32. 10.1097/j.jcrs.0000000000000317 32657904

[8] Frolov MA Frolov AM Kazakova KA. [Combination treatment for cataract and glaucoma]. Vestn Oftalmol. (2017) 133:42–6. 10.17116/oftalma2017133442-46 28980565

[9] Han X Fan Q Hua Z Qiu X Qian D Yang J. Analysis of corneal astigmatism and aberration in Chinese congenital cataract and developmental cataract patients before cataract surgery. BMC Ophthalmol. (2021) 21:34. 10.1186/s12886-020-01794-2 33435913 PMC7805192

[10] Tan S Chen X Cui C Hou Y Li W You H. Biodegradation of saline phenolic wastewater in a biological contact oxidation reactor with immobilized cells of *Oceanimonas* sp. Biotechnol Lett. (2017) 39:91–6. 10.1007/s10529-016-2226-9 27659032

[11] Charles Crozafon P Bouchet C Zignani M Griner R Foster SD Zou M. Comparison of real-world treatment outcomes of femtosecond laser-assisted cataract surgery and phacoemulsification cataract surgery: A retrospective, observational study from an outpatient clinic in France. Eur J Ophthalmol. (2021) 31:1809–16. 10.1177/1120672120925766 32452248

[12] Foster GJL Allen QB Ayres BD Devgan U Hoffman RS Khandelwal SS. Phacoemulsification of the rock-hard dense nuclear cataract: options and recommendations. J Cataract Refract Surg. (2018) 44:905–16. 10.1016/j.jcrs.2018.03.038 29960655

[13] Maehara S Matsumoto N Takiyama N Itoh Y Kitamura Y Yamashita K. Surgical removal of cataract in an Asiatic black bear (*Ursus thibetanus*) by phacoemulsification and aspiration. J Vet Med Sci. (2020) 82:740–4. 10.1292/jvms.19-0639 32295988 PMC7324814

[14] Qian Z Huang J Song B Wei L Fu L Austin MW. Cataract surgery (Phacoemulsification with Intraocular Lens Implantation) combined with endoscopic goniosynechialysis for advanced primary angle-closure glaucoma. Ophthalmol Glaucoma. (2021) 4:365–72. 10.1016/j.ogla.2020.11.003 33242682

[15] Han JV Patel DV Liu K Kim BZ Sherwin T McGhee CNJ. Auckland cataract study IV: practical application of NZCRS cataract risk stratification to reduce phacoemulsification complications. Clin Exp Ophthalmol. (2020) 48:311–8. 10.1111/ceo.13696 31804765

[16] He L Cui Y Tang X He S Yao X Huang Q. Changes in visual function and quality of life in patients with senile cataract following phacoemulsification. Ann Palliat Med. (2020) 9:3802–9. 10.21037/apm-20-1709 33183034

[17] Kozera M Konopińska J Mariak Z Rekas M. Effectiveness of iStent trabecular microbypass system combined with phacoemulsification versus phacoemulsification alone in patients with glaucoma and cataract depending on the initial intraocular pressure. Ophthalmic Res. (2021) 64:327–36. 10.1159/000511456 32906138

[18] Nyström A Almarzouki N Magnusson G Zetterberg M. Phacoemulsification and primary implantation with bag-in-the-lens intraocular lens in children with unilateral and bilateral cataract. Acta Ophthalmol. (2018) 96:364–70. 10.1111/aos.13626 29350795

[19] Cairns JE. Trabeculectomy. Preliminary report of a new method. Am J Ophthalmol. (1968) 66:673–9. 10.1016/0002-9394(68)91288-9 4891876

[20] Hansapinyo L Choy BNK Lai JSM Tham CC. Phacoemulsification versus phacotrabeculectomy in primary angle-closure glaucoma with cataract: long-term clinical outcomes. J Glaucoma. (2020) 29:15–23. 10.1097/IJG.0000000000001397 31702714

[21] Rao A Padhy D Das G Sarangi S. Viscoless manual small incision cataract surgery with trabeculectomy. Semin Ophthalmol. (2018) 33:552–9. 10.1080/08820538.2017.1339092 28665780

[22] Elwehidy AS Bayoumi NHL Badawi AE Hagras SM Kamel R. Combined phacoemulsification-viscosynechialysis-trabeculotomy vs phacotrabeculectomy in uncontrolled primary angle-closure glaucoma with cataract. J Cataract Refract Surg. (2019) 45:1738–45. 10.1016/j.jcrs.2019.07.031 31856984

[23] Anbar M Ammar H. Effect of different incision sites of phacoemulsification on trabeculectomy bleb function: prospective case-control study. BMC Ophthalmol. (2017) 17:103. 10.1186/s12886-017-0500-9 28651590 PMC5485683

[24] Wang Y Liang ZQ Zhang Y Hennein L Han Y Wu HJ. Efficacy and safety of phacoemulsification plus goniosynechialysis and trabectome in patients with primary angle-closure glaucoma. Sci Rep. (2021) 11:13921. 10.1038/s41598-021-92972-9 34230569 PMC8260581

[25] Choi SO Jeon HS Hyon JY Oh YJ Wee WR Chung TY. Recovery of corneal endothelial cells from periphery after injury. PLoS ONE. (2015) 10:e0138076. 10.1371/journal.pone.0138076 26378928 PMC4574742

